# Antimicrobial Resistance Patterns Among Gram-Negative Bacterial Isolates Recovered from Tracheal Aspirate Cultures in Türkiye: A Systematic Review and Meta-Analysis

**DOI:** 10.3390/biomedicines14102196

**Published:** 2026-09-28

**Authors:** Elmas Pinar Kahraman Kilbas, Ihsan Hakki Ciftci, Imdat Kilbas, Tamar Didbaridze

**Affiliations:** 1 Medical Laboratory Techniques Program, Vocational School of Health Services, Fenerbahce University, Istanbul 34758, Türkiye; elmspnrkk@gmail.com; 2 Department of Medical Microbiology, Faculty of Medicine, Sakarya University, Sakarya 54100, Türkiye; 3 Dental Prosthesis Technology Program, Vocational School of Health Services, Fenerbahce University, Istanbul 34758, Türkiye; imdtklbs@gmail.com; 4 Department of Microbiology, Tbilisi State Medical University, Tbilisi 0186, Georgia

**Keywords:** *Acinetobacter baumannii*, *Pseudomonas aeruginosa*, *Klebsiella pneumoniae*, drug resistance, tracheal aspirate, prevalence

## Abstract

**Background:** This systematic review and meta-analysis aimed to evaluate antimicrobial resistance patterns among selected Gram-negative bacterial isolates recovered from tracheal aspirate cultures in Türkiye. **Methods:** The systematic review was conducted in accordance with the Preferred Reporting Items for Systematic Reviews and Meta-Analyses (PRISMA) guidelines. Electronic databases were systematically searched to identify eligible studies reporting antimicrobial resistance patterns among selected Gram-negative bacterial isolates from tracheal aspirate cultures in Türkiye. Random-effects meta-analyses of study-level resistance proportions were performed using the number of resistant and tested isolates reported by each study. Between-study heterogeneity was assessed using Cochran’s Q test and the I^2^ statistic. Sensitivity analyses and exploratory univariable meta-regression analyses were also performed. **Results:** Eighteen studies comprising 5436 isolates belonging to five selected Gram-negative bacterial groups recovered from tracheal aspirate cultures were included. The pooled meropenem resistance rates were 93.21% for *A. baumannii*, 37.7% for *P. aeruginosa*, 29.3% for *K. pneumoniae*, and 4.3% for *E. coli*. *Pseudomonas aeruginosa* showed a higher pooled meropenem resistance estimate than *K. pneumoniae*, while both species exhibited substantial resistance to several other commonly used antimicrobial agents. Exploratory meta-regression indicated an increase in meropenem resistance across more recent study periods for *K. pneumoniae*, whereas no significant temporal trend was observed for *A. baumannii* or *P. aeruginosa*. **Conclusions:** Multidrug-resistant Gram-negative bacteria continue to represent a major problem in tracheal aspirate cultures in Türkiye. In particular, the high prevalence of carbapenem resistance in *A. baumannii* highlights the need for continuous national surveillance, standardized microbiological methods, strengthened antimicrobial stewardship, and effective infection prevention and control measures.

## 1. Introduction

Healthcare-associated infections (HAIs) are infections that occur during the course of a patient’s care in a hospital or healthcare facility and are neither present nor in the incubation period at the time of hospital admission [1].

According to reports published by the European Centre for Disease Prevention and Control, HAIs occurring in intensive care units account for a large proportion of hospital-acquired infections in Europe [2]. Ventilator-associated pneumonia, bloodstream infections, and catheter-associated urinary tract infections are the most common HAIs. These infections prolong hospital stays, increase healthcare costs, and result in productivity losses [3]. According to the Global Burden of Disease Study 2023, approximately 2.5 million people died worldwide from lower respiratory tract infections in 2023 [4].

Tracheostomy is performed to provide an open airway in patients who are unable to protect their airway and to facilitate weaning from mechanical ventilation. However, due to the loss of upper airway function, it may increase the risk of microbial colonization and infection. The literature has reported that tracheostomy increases the risk of developing ventilator-associated pneumonia [5].

Due to the presence of antimicrobial-resistant microorganisms, broad-spectrum antibiotics are frequently used in intensive care units. In particular, pneumonia caused by multidrug-resistant Gram-negative bacteria has become increasingly common, reflecting a shift toward Gram-negative etiologies and the rapid dissemination of these pathogens in hospital settings [6].

Antimicrobial resistance among healthcare-associated Gram-negative bacteria poses a significant problem in Türkiye, particularly in intensive care units. National surveillance data from 2022 reported carbapenem resistance rates of 92.2% for *A. baumannii*, 67.6% for *P. aeruginosa*, and 66.6% for *K. pneumoniae*. The high prevalence of carbapenem-resistant isolates, combined with the endemic circulation of OXA-48-like and other carbapenemases, highlights the serious regional burden of antimicrobial resistance and the need for continuous surveillance [7,8].

Therefore, the accurate identification of causative microorganisms and the determination of antibiotic resistance profiles in infections developing in intensive care units play a critical role in the effective management of healthcare-associated infections and the control of antibiotic resistance.

The aim of this study was to systematically evaluate antimicrobial resistance patterns among Gram-negative bacterial isolates recovered from tracheal aspirate cultures in Türkiye, while considering differences in microbiological methods and reporting standards among the included studies.

## 2. Materials and Methods

### 2.1. Study Design

This systematic review and meta-analysis evaluated antimicrobial resistance patterns among selected Gram-negative bacterial isolates recovered from tracheal aspirate cultures in Türkiye. The study design was based on the internationally recognized PRISMA (Preferred Reporting Items for Systematic Reviews and Meta-Analyses) guidelines for systematic reviews and meta-analyses [9]. The review was registered retrospectively in the PROSPERO database (CRD420261471090) on 5 August 2026. Following registration, the scope was refined to focus on antimicrobial resistance among selected Gram-negative bacterial isolates; this change was recorded as a protocol deviation. The PRISMA checklist is presented in Table S1.

### 2.2. Literature Search Strategy

The systematic literature search was conducted in the PubMed/MEDLINE, Embase, Web of Science, Scopus, Turk Medline, and Google Scholar databases for studies published until 1 December 2025. The final database search was conducted on 2 April 2026. The following databases and platforms were searched: MEDLINE via PubMed, Embase via Embase.com, Web of Science Core Collection, Scopus, Turk Medline, and Google Scholar. Google Scholar was used as a supplementary search source, and the first 200 results ranked by relevance were screened. The search was performed using the keywords “tracheal aspirate”, “endotracheal aspirate”, “bacterial isolates”, “pathogens”, “antimicrobial resistance”, “antibiotic resistance”, “antimicrobial susceptibility”, “drug resistance”, “MIC”, “susceptibility”, “prevalence”, and “Turkey”, as well as combinations of these terms (Table A1). No manual search or snowballing strategy was performed in addition to the predefined electronic database search. No database filters were applied during the searches. Eligibility was subsequently restricted to articles published in English or Turkish. Backward and forward citation tracking and reference-list screening were not performed.

### 2.3. Inclusion and Exclusion Criteria

The analysis included studies conducted in Türkiye reporting Gram-negative bacterial isolates obtained from tracheal aspirate samples. Only original research articles published in English or Turkish were considered. Studies reporting bacterial species identified from tracheal aspirate cultures and providing antimicrobial susceptibility or resistance data for the isolates were deemed eligible for inclusion. In addition, studies conducted on human clinical samples and providing sufficient microbiological data for analysis were also included.

Studies that did not specifically report bacterial isolates obtained from tracheal aspirate samples were excluded. Theses, reviews, meta-analyses, and book chapters were also excluded. Studies that did not report bacterial species individually or did not provide antimicrobial susceptibility data were also excluded. Publications written in languages other than English or Turkish were not considered. In addition, studies not conducted in Türkiye, duplicate publications, studies whose full text was not accessible, experimental studies not conducted on human samples, and studies with insufficient microbiological data were also excluded.

The included studies inconsistently reported sampling indications, diagnostic criteria, culture thresholds, and handling of repeated isolates. Therefore, colonization could not be reliably distinguished from clinically confirmed infection.

### 2.4. Study Selection and Data Extraction

After independently reviewing titles and abstracts, two authors evaluated the full texts of potentially eligible studies. Disagreements were resolved through discussion and consensus; a third author was consulted when consensus could not be reached.

Data were independently extracted by two authors using a standardized form. Extracted variables included the year of publication, study location, methods for bacterial identification and antimicrobial susceptibility testing, interpretation guidelines, the number of eligible Gram-negative isolates, the number of isolates tested for each antimicrobial agent, and antimicrobial resistance results. The number of tracheal aspirate samples examined and the number of bacterial isolates obtained were extracted and recorded separately. The unit of analysis used in the resistance meta-analyses was the number of bacterial isolates. In cases where the total number of tracheal aspirate samples was not reported in the primary study, these data were recorded as “not reported”. Where available, indications for sampling, diagnostic criteria, culture thresholds, and the handling of repeat isolates were also recorded. Discrepancies were resolved through consensus.

### 2.5. Risk of Bias Assessment

The risk of bias of the included studies was evaluated using the Joanna Briggs Institute (JBI) Prevalence Studies Checklist. Each study was classified as having a low, moderate, or high risk of bias. Risk of bias assessment was performed independently by two researchers. Studies with a JBI assessment score below four were excluded [10]. Studies scoring 7–9 were classified as low risk, those scoring 4–6 as moderate risk, and those scoring below 4 as high risk and were excluded. According to this evaluation, 7 studies were rated as moderate risk and 11 studies were rated as being at low risk of bias.

### 2.6. Statistical Analysis

All analyses were performed using R version 4.3.3 and the metafor package version 4.4–0 [11]. Study-level resistance proportions were transformed using the Freeman–Tukey double-arcsine transformation [12]. Random-effects models were fitted using inverse-variance weighting, and between-study variance was estimated using the DerSimonian–Laird method. Heterogeneity was assessed using Cochran’s Q, I^2^, and τ^2^. Pooled estimates and 95% confidence intervals were back-transformed using the Miller method and the harmonic mean of the study-specific denominators [13]. Total isolate counts presented in Table 2 are descriptive and were not used as a single aggregated denominator. Forest plots were presented on the original proportion scale. The complete R code and analytical dataset are provided in Supplementary Files S1 and S2. Leave-one-out sensitivity analyses were performed for the primary meropenem analyses. Meta-regression analyses were conducted to examine study year and antimicrobial susceptibility interpretation guideline as potential moderators. For the temporal meta-regression, year was defined as the midpoint of the study period rather than the publication year. For studies conducted within a single calendar year, that year was used directly. Because the versions of the susceptibility interpretation guidelines were not consistently reported, resistance classifications could not be retrospectively harmonized across all studies. Therefore, only isolates explicitly reported as resistant by the original studies were included in the resistance numerator. 

## 3. Results

### 3.1. Study Characteristics

A total of 1756 records were identified through database searching. After removal of duplicate records and screening according to the predefined exclusion criteria, 277 full-text articles were assessed for eligibility, and 18 original research articles were ultimately included in the study (Figure 1).

The 18 included studies contributed a total of 5436 isolates belonging to the five selected Gram-negative bacterial groups recovered from tracheal aspirate cultures. This number represents the eligible bacterial isolates included in the review and not the number of tracheal aspirate specimens. The total number of tracheal aspirate specimens across all studies could not be calculated because this information was not reported in several primary studies. The analysis included *A. baumannii*, *P. aeruginosa*, *K. pneumoniae*, *E. coli*, and *Enterobacter* spp. The studies used various bacterial identification and antimicrobial susceptibility testing methods, most commonly VITEK 2, conventional methods, and BD Phoenix systems, in accordance with CLSI and/or EUCAST guidelines. The main characteristics of the included studies are summarized in Table 1 and Table S2.

### 3.2. Antimicrobial Resistance Patterns and Meta-Analysis

The pooled resistance rates of the major Gram-negative bacterial isolates recovered from tracheal aspirate samples are presented in Table 2. *Acinetobacter baumannii* exhibited the highest pooled meropenem resistance rate at 93.21%, followed by *P. aeruginosa* at 37.65%, *K. pneumoniae* at 29.26%, and *E. coli* at 4.25%. *Enterobacter* spp. results were not pooled because species-level identification was inconsistently reported.

Because carbapenem resistance represents an important therapeutic challenge in healthcare-associated Gram-negative infections, meropenem resistance was selected for detailed graphical presentation. Separate random-effects analyses were presented for *A. baumannii*, *P. aeruginosa*, *K. pneumoniae*, and *E. coli*. Forest plots display study-specific and pooled resistance estimates on the original proportion scale.

The pooled meropenem resistance rate was 93.21% (95% CI: 90.44–95.57%) for *A. baumannii* based on 13 studies, with substantial heterogeneity (Q = 46.16, df = 12, *p* < 0.001; I^2^ = 74.0%; τ^2^ = 0.005215). The corresponding estimate was 37.65% (95% CI: 25.87–50.18%) for *P. aeruginosa* based on 10 studies (Q = 91.59, df = 9, *p* < 0.001; I^2^ = 90.2%; τ^2^ = 0.033261) and 29.26% (95% CI: 15.84–44.68%) for *K. pneumoniae* based on nine studies (Q = 104.91, df = 8, *p* < 0.001; I^2^ = 92.4%; τ^2^ = 0.049559). A lower pooled estimate was observed for *E. coli* (4.25%; 95% CI: 0.03–12.34%; six studies; I^2^ = 72.8%; τ^2^ = 0.017117) (Figure 2).

The distribution of the study-specific estimates and the heterogeneity statistics indicated considerable between-study variability, particularly for *P. aeruginosa*, *K. pneumoniae*, and *E. coli*. Leave-one-out analyses did not identify a single study whose exclusion qualitatively changed the principal findings; however, substantial heterogeneity remained.

### 3.3. Meta-Regression Analysis

Univariable meta-regression analyses were conducted to examine the midpoint of the study period and antimicrobial susceptibility guideline as potential moderators of meropenem resistance. Study mid-year was not significantly associated with meropenem resistance in *A. baumannii* (*p* = 0.072) or *P. aeruginosa* (*p* = 0.322). In contrast, a positive association was observed for *K. pneumoniae* (*p* = 0.024), suggesting an increase in meropenem resistance across more recent study periods. Guideline category was significantly associated with meropenem resistance in *A. baumannii* (*p* = 0.002). Residual heterogeneity remained substantial in most models (Table 3). Because some models included fewer than 10 studies, the meta-regression findings were considered exploratory and interpreted cautiously.

## 4. Discussion

This systematic review and meta-analysis provides an overview of antimicrobial resistance patterns among selected Gram-negative bacterial isolates recovered from tracheal aspirate cultures in Türkiye over approximately two decades. High resistance rates were observed among these isolates, particularly against carbapenems and several commonly used antimicrobial agents. However, because the source studies did not consistently distinguish colonization from clinically confirmed infection, these findings should not be interpreted as resistance patterns exclusively among causative respiratory pathogens. These findings are consistent with international surveillance reports indicating that multidrug-resistant Gram-negative bacterial species remain among the most important threats to patient safety in intensive care units, where mechanical ventilation, prolonged hospitalization, invasive procedures, and intensive antibiotic exposure facilitate both pathogen transmission and the selection of resistant organisms [32,33].

Among all evaluated bacterial species, *A. baumannii* exhibited the highest resistance rates, particularly to meropenem and imipenem. The pooled meropenem resistance rate was 93.21%, indicating markedly limited in vitro activity of meropenem against *A. baumannii* isolates and highlighting the importance of local susceptibility data when interpreting resistance patterns among clinical isolates. This observation is consistent with the World Health Organization, which classifies carbapenem-resistant *A. baumannii* isolates among the highest-priority bacterial pathogens requiring the urgent development of new antimicrobial strategies [32].

Resistance in *A. baumannii* generally results from multiple overlapping mechanisms rather than a single genetic event. These include the production of OXA-type carbapenemases (particularly OXA-23, OXA-24/40, and OXA-58), metallo-β-lactamases such as New Delhi metallo-β-lactamase (NDM), reduced outer membrane permeability, alterations in penicillin-binding proteins, and overexpression of multidrug efflux pumps [34]. The coexistence of multiple resistance mechanisms within the same isolate frequently leads to extensively drug-resistant (XDR) phenotypes, explaining why resistance to meropenem, imipenem, fluoroquinolones, and aminoglycosides commonly occurs simultaneously [35,36]. In recent years, an increasing prevalence of XDR strains among *A. baumannii* isolates has been reported [37].

*Pseudomonas aeruginosa* and *K. pneumoniae* exhibited lower pooled meropenem resistance rates than *A. baumannii*, with pooled estimates of 37.65% and 29.26%, respectively. These findings indicate that carbapenem resistance is not limited to *A. baumannii* but also represents a significant therapeutic challenge among other major Gram-negative isolates in intensive care units. Similar epidemiological patterns have also been reported by the European Antimicrobial Resistance Surveillance Network, which has documented increasing rates of carbapenem-resistant *P. aeruginosa* and *Enterobacterales* in many European countries in recent years [38].

Beyond carbapenem resistance, *K. pneumoniae* exhibited resistance to commonly used antimicrobial classes, further limiting the available treatment options. Türkiye has long been recognized as an endemic region for OXA-48-producing *K. pneumoniae*, and the continued circulation of OXA-48, NDM, and *Klebsiella pneumoniae* carbapenemase (KPC)-producing isolates, often accompanied by porin loss and extended-spectrum β-lactamase production, has likely contributed to the resistance patterns observed in the present analysis [39,40]. Exploratory meta-regression showed a positive association between study mid-year and meropenem resistance in *K. pneumoniae*, suggesting that resistance estimates were higher in studies conducted during more recent periods. This finding is consistent with the increasing dissemination of carbapenemase-producing *K. pneumoniae* lineages, including OXA-48-like-, NDM-, and KPC-producing strains. However, the analysis was based on only nine studies and residual heterogeneity remained high. Therefore, this association should be interpreted cautiously and should not be considered definitive evidence of a national temporal trend.

Although carbapenem resistance in *P. aeruginosa* was lower than that observed in *A. baumannii*, resistance remained substantial. Resistance mechanisms such as AmpC β-lactamase overexpression, OprD porin loss, activation of multidrug efflux pumps, and acquisition of carbapenemases contribute to the persistence and antimicrobial resistance of *P. aeruginosa*, a recognized cause of lower respiratory tract infections in critically ill patients [41]. The coexistence of these resistance mechanisms is associated with increasingly limited treatment options, particularly in critically ill patients requiring mechanical ventilation. Accordingly, the substantial carbapenem resistance observed among the included isolates underscores the importance of considering local antimicrobial susceptibility data when interpreting tracheal aspirate culture results. Nevertheless, culture results should be evaluated together with clinical and radiological findings before treatment decisions are made [42].

In contrast, *E. coli* exhibited a lower pooled meropenem resistance estimate of 4.25%. However, this finding should be interpreted cautiously because it was based on only six studies and was accompanied by substantial heterogeneity. Larger multicenter surveillance studies using standardized microbiological methods are needed to characterize antimicrobial resistance patterns in *E. coli* more reliably.

The high antimicrobial resistance rates observed in tracheal aspirate cultures in this study may be associated with several epidemiological factors that facilitate the dissemination of multidrug-resistant Gram-negative bacteria in intensive care units. Critically ill patients requiring mechanical ventilation are frequently exposed to prolonged hospitalization, repeated invasive procedures, broad-spectrum antimicrobial therapy, and the use of invasive medical devices. These conditions create strong selective pressure that facilitates the selection of resistant bacteria and the transmission of healthcare-associated infections. Furthermore, the ability of *A. baumannii* and *P. aeruginosa* to survive for prolonged periods on hospital surfaces and medical equipment represents an important characteristic that facilitates their nosocomial spread despite infection control measures [35,43].

A major finding of this meta-analysis was the substantial between-study heterogeneity observed in the pooled antimicrobial resistance estimates. Such heterogeneity is expected in prevalence meta-analyses because antimicrobial resistance is influenced by local epidemiology, antimicrobial prescribing practices, patient populations, infection prevention policies, and microbiological laboratory methodologies. While most studies used automated antimicrobial susceptibility testing systems such as VITEK 2 or BD Phoenix, others relied on conventional methods. Similarly, susceptibility interpretation criteria differed between the CLSI and EUCAST guidelines. Previous studies have shown that differences in susceptibility breakpoints, particularly for carbapenems and β-lactam antibiotics, may substantially influence reported resistance rates [44]. In the present exploratory meta-regression, guideline category was associated with meropenem resistance estimates for *A. baumannii* but not for *P. aeruginosa* or *K. pneumoniae*. The association observed for *A. baumannii* should be interpreted cautiously because guideline use may have been confounded by study period, institution, patient population, laboratory method, and breakpoint version. Substantial residual heterogeneity, particularly in the *P. aeruginosa* and *K. pneumoniae* models, indicates that guideline category explained only a limited portion of the variability between studies. Therefore, the pooled estimates should be interpreted as descriptive resistance patterns among Gram-negative isolates recovered from tracheal aspirate cultures rather than as national infection rates or universally applicable resistance rates for individual healthcare institutions. The substantial methodological heterogeneity observed across the included studies highlights the need for standardized microbiological methodologies to generate more reliable and comparable antimicrobial resistance data. This would strengthen microbiological surveillance and improve the interpretation of antimicrobial resistance data from tracheal aspirate cultures.

This study has several strengths. To the best of our knowledge, this is the first systematic review and meta-analysis specifically evaluating antimicrobial resistance patterns among selected Gram-negative bacterial isolates in tracheal aspirate cultures from Türkiye. By integrating data from multiple centers over an extended period, this analysis provides an overview of antimicrobial resistance patterns among selected Gram-negative isolates recovered from tracheal aspirate cultures. Furthermore, the use of a standardized systematic review methodology, independent study selection, risk of bias assessment, and quantitative meta-analysis enhances the robustness and reliability of the findings.

Several limitations should be considered. Most included studies were retrospective and conducted in intensive care units, which may limit the generalizability of the findings. Antimicrobial susceptibility methods and reporting varied across studies, contributing to heterogeneity and limiting some pooled analyses. Moreover, the studies did not consistently report clinical diagnostic criteria, culture thresholds, or the handling of repeated isolates; therefore, airway colonization could not be reliably distinguished from respiratory infection. The findings should thus be interpreted as resistance patterns among recovered isolates rather than confirmed causative pathogens. The versions of the CLSI and EUCAST guidelines were not consistently reported in the included studies; therefore, resistance results could not be retrospectively harmonized according to a single breakpoint standard. Changes in breakpoints and susceptibility categories over time may have introduced misclassification and contributed to between-study heterogeneity. Finally, formal tests of funnel plot asymmetry were not performed because their interpretation may be limited by the small number of studies and substantial heterogeneity.

## 5. Conclusions

In conclusion, high antimicrobial resistance rates, particularly to carbapenems, were observed among selected Gram-negative bacterial isolates recovered from tracheal aspirate cultures in Türkiye. *A. baumannii* exhibited the highest carbapenem resistance rates among the evaluated species. These findings describe resistance patterns among the selected isolates and should not be interpreted as a comprehensive assessment of bacterial distribution or as evidence of clinically confirmed respiratory infection. Future multicenter studies using standardized microbiological and clinical criteria are needed to improve the comparability of antimicrobial resistance data.

## Figures and Tables

**Figure 1:**
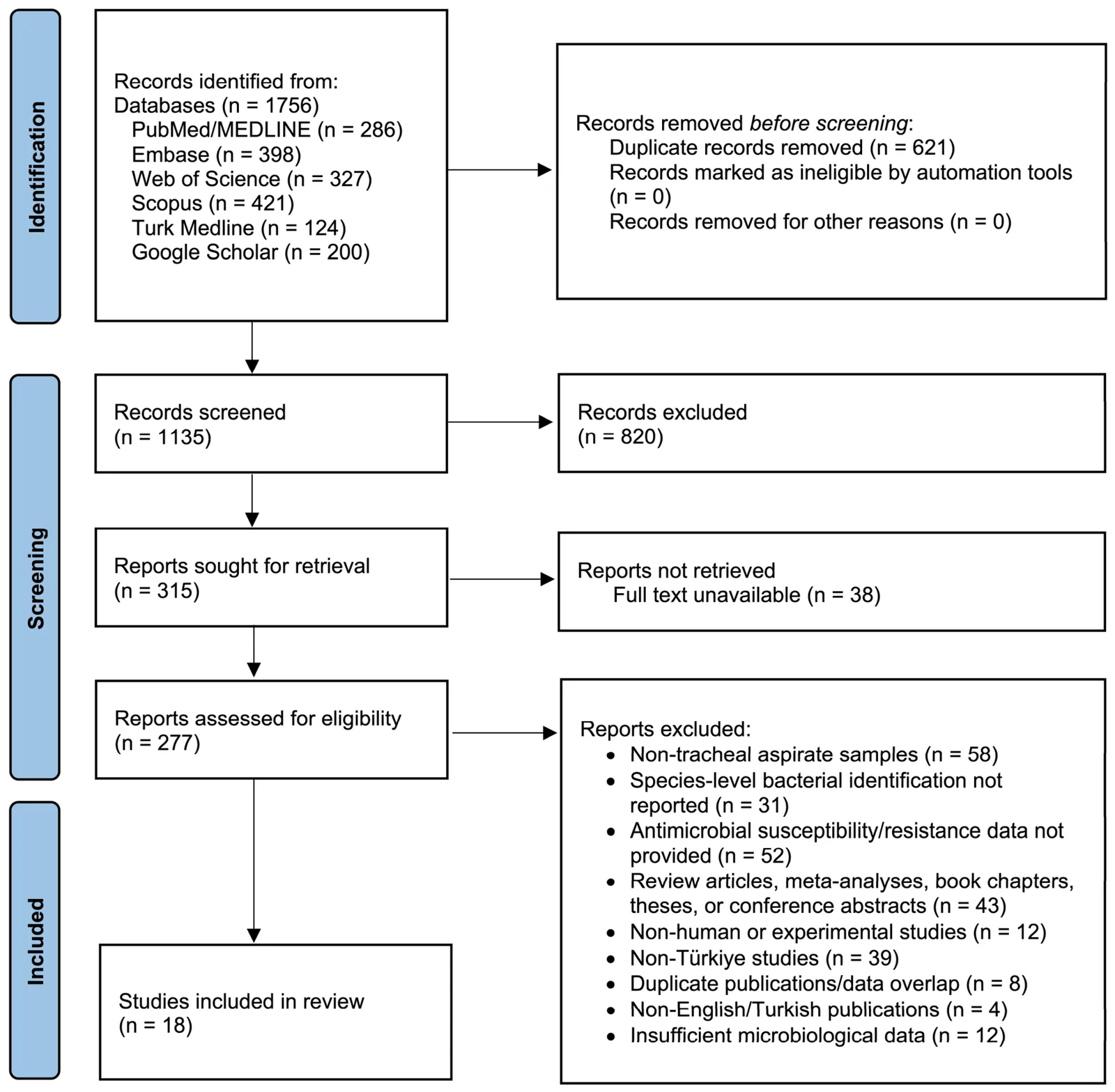
PRISMA flow diagram of study identification, screening, eligibility assessment, and inclusion.

**Figure 2:**
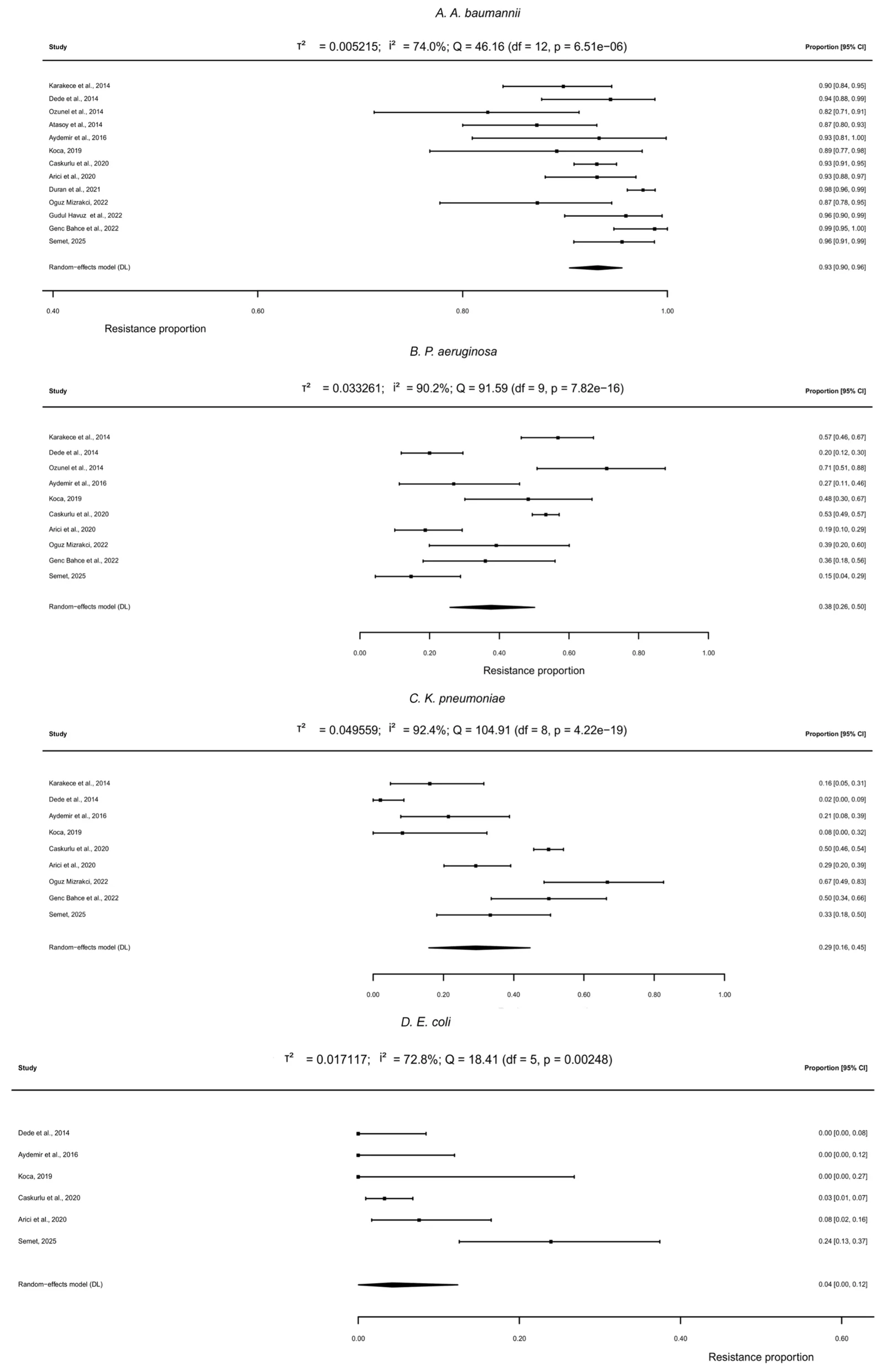
Forest plots of study-specific and random-effects pooled meropenem resistance proportions for (**A**) *A. baumannii*, (**B**) *P. aeruginosa*, (**C**) *K. pneumoniae*, and (**D**) *E. coli*. Study-specific estimates and 95% confidence intervals are presented on the original proportion scale. Diamonds represent the pooled estimates and their 95% confidence intervals [14,15,16,17,18,19,20,21,22,23,24,25,26,27,28,29,30,31].

**Table 1:** Characteristics of included studies [14,15,16,17,18,19,20,21,22,23,24,25,26,27,28,29,30,31].

Author	Study Period	City	Identification Method	Antimicrobial Susceptibility Testing Method	Guideline	Tracheal Aspirate Specimens, *n*	Eligible Target Gram-Negative Isolates Included, *n*	ICU Type	JBI Assessment
Safak et al., 2007 [14]	2004–2006	Afyonkarahisar	CM	mini-API and Phoenix	CLSI; version NR	455	128	Mixed	Moderate
Ulug et al., 2011 [15]	2003–2005	Diyarbakir Formun Üstü Formun Altı	CM	KB/DD	NCCLS M2-A7	NR	54	Mixed	Moderate
Kume and Demirci, 2012 [16]	2008–2010	Izmir	CM	KB/DD	CLSI M100-S18 (2008)	NR	175	Mixed	Moderate
Atasoy et al., 2014 [17]	2012–2013	Sakarya	VITEK 2	VITEK 2	CLSI; version NR	NR	102	General	Moderate
Dede et al., 2014 [18]	2010–2011	Istanbul	VITEK 2	VITEK 2	CLSI; version NR	192	220	General	Moderate
Karakece et al., 2014 [19]	2011–2012	Sakarya	VITEK 2	VITEK 2	CLSI M100-S20 (2010)	856	257	General	Low
Ozunel et al., 2014 [20]	2012–2013	Corum	VITEK 2	VITEK 2	CLSI M100-S22 (2012)	238	81	Anesthesia	Moderate
Aydemir et al., 2016 [21]	2015–2016	Sakarya	VITEK 2	VITEK 2	CLSI 2015; document number NR	350	110	Mixed	Low
Sagmak-Tartar et al., 2018 [22]	2016–2017	Elazig	VITEK 2	KB/DD	CLSI; version NR	1470	553	Mixed	Low
Koca, 2019 [23]	2017–2018	Antalya	VITEK 2	VITEK 2	EUCAST; version NR	205	88	Adult	Low
Arici et al., 2020 [24]	2016–2018	Istanbul	MALDI-TOF MS	VITEK 2	EUCAST; version NR	NR	337	General	Low
Caskurlu et al., 2020 [25]	2014–2018	Istanbul	VITEK 2	VITEK 2	CLSI M100-S24/S25/S26 (2014–2016); annual EUCAST criteria (2017–2018)	5201	1907	Mixed	Low
Duran et al., 2021 [26]	2015–2019	Tekirdag	BD Phoenix	BD Phoenix	EUCAST v9.0 (2019)	NR	505	Mixed	Moderate
Ayvalik et al., 2022 [27]	2018–2021	Isparta	BD Phoenix	BD Phoenix	EUCAST breakpoint tables 2020; CLSI M100 Ed30 (2020)	1031	356	General	Low
Genc Bahce et al., 2022 [28]	2019–2020	Siirt	VITEK 2	VITEK 2	EUCAST; version NR	NR	142	COVID-19/General	Low
Gudul Havuz et al., 2022 [29]	2019–2020	Samsun	VITEK 2	VITEK 2	EUCAST; version NR	NR	74	Mixed	Low
Oguz Mizrakci, 2022 [30]	2019–2020	Antalya	CM	KB/DD and	CLSI M100 (2016)	288	116	General	Low
Semet, 2025 [31]	2022	Bursa	CM	E-test	EUCAST; version NR	NR	231	General	Low

Specimen and isolate counts represent different quantities. More than one isolate could be recovered from a single tracheal aspirate specimen. The eligible isolate count includes only isolates belonging to the five Gram-negative bacterial groups evaluated in this review. CM: conventional methods; KB/DD: Kirby–Bauer disk diffusion; ICU: intensive care unit; NR: not reported.

**Table 2:** Random-effects pooled antimicrobial resistance estimates for Gram-negative bacterial isolates recovered from tracheal aspirate cultures.

Pathogen	Drug	k	Total Tested	Pooled Resistance (95% CI)	τ^2^	I^2^	Q (*p*)
*A. baumannii*	IPM	17	2525	85.72% (78.50–91.74%)	0.033438	94.9%	311.86 (*p* < 0.001)
*A. baumannii*	MEM	13	1973	93.21% (90.44–95.57%)	0.005215	74.0%	46.16 (*p* < 0.001)
*A. baumannii*	AMK	18	2638	59.39% (48.92–69.46%)	0.046234	96.2%	448.72 (*p* < 0.001)
*A. baumannii*	GEN	14	2144	82.34% (73.44–89.80%)	0.037240	95.4%	283.85 (*p* < 0.001)
*A. baumannii*	CIP	16	2509	93.32% (90.00–96.06%)	0.010287	85.7%	105.18 (*p* < 0.001)
*A. baumannii*	LEV	3	175	91.72% (83.88–97.29%)	0.006060	57.6%	4.72 (*p* = 0.094)
*A. baumannii*	SXT	14	2162	75.88% (66.07–84.52%)	0.037311	95.5%	287.17 (*p* < 0.001)
*P. aeruginosa*	IPM	14	1326	40.10% (29.68–50.96%)	0.035756	91.6%	154.20 (*p* < 0.001)
*P. aeruginosa*	MEM	10	1034	37.65% (25.87–50.18%)	0.033261	90.2%	91.59 (*p* < 0.001)
*P. aeruginosa*	AMK	15	1360	28.88% (17.08–42.26%)	0.066930	95.2%	289.65 (*p* < 0.001)
*P. aeruginosa*	CAZ	12	1273	45.83% (37.01–54.77%)	0.018996	86.4%	80.89 (*p* < 0.001)
*P. aeruginosa*	TZP	11	1173	56.40% (46.68–65.89%)	0.020670	86.8%	75.85 (*p* < 0.001)
*P. aeruginosa*	CIP	13	1256	43.40% (30.48–56.77%)	0.052220	94.0%	200.44 (*p* < 0.001)
*P. aeruginosa*	LEV	4	104	41.74% (27.10–57.09%)	0.013511	58.8%	7.29 (*p* = 0.063)
*K. pneumoniae*	IPM	11	959	25.56% (10.42–44.13%)	0.091414	95.9%	244.35 (*p* < 0.001)
*K. pneumoniae*	MEM	9	840	29.26% (15.84–44.68%)	0.049559	92.4%	104.91 (*p* < 0.001)
*K. pneumoniae*	AMK	13	1028	40.58% (25.20–56.89%)	0.075042	94.8%	231.25 (*p* < 0.001)
*K. pneumoniae*	GEN	9	859	40.65% (30.59–51.11%)	0.018012	82.2%	45.00 (*p* < 0.001)
*K. pneumoniae*	CAZ	10	935	66.47% (59.60–73.03%)	0.006372	63.3%	24.53 (*p* = 0.004)
*K. pneumoniae*	TZP	10	966	65.86% (55.56–75.49%)	0.021624	86.2%	65.12 (*p* < 0.001)
*K. pneumoniae*	CIP	12	980	60.86% (46.97–73.97%)	0.047374	92.0%	137.84 (*p* < 0.001)
*K. pneumoniae*	SXT	10	887	66.46% (54.56–77.45%)	0.028122	87.3%	71.09 (*p* < 0.001)
*E. coli*	IPM	9	317	1.01% (0.00–3.94%)	0.002944	25.5%	10.74 (*p* = 0.217)
*E. coli*	MEM	6	292	4.25% (0.03–12.34%)	0.017117	72.8%	18.41 (*p* = 0.002)
*E. coli*	AMK	10	363	24.74% (11.62–40.41%)	0.051753	86.7%	67.84 (*p* < 0.001)
*E. coli*	GEN	7	293	30.29% (17.42–44.75%)	0.025215	77.2%	26.27 (*p* < 0.001)
*E. coli*	CAZ	7	291	54.53% (40.23–68.50%)	0.023116	75.4%	24.38 (*p* < 0.001)
*E. coli*	TZP	8	337	31.67% (26.55–37.00%)	0.000000	0.0%	6.35 (*p* = 0.499)
*E. coli*	CIP	9	343	51.51% (39.63–63.30%)	0.018212	70.3%	26.94 (*p* < 0.001)
*E. coli*	SXT	8	343	38.68% (28.03–49.85%)	0.014981	68.9%	22.51 (*p* = 0.002)

IPM: imipenem; MEM: meropenem; AMK: amikacin; GEN: gentamicin; CIP: ciprofloxacin; LEV: levofloxacin; SXT: trimethoprim–sulfamethoxazole; CAZ: ceftazidime; TZP: piperacillin–tazobactam; CI: confidence interval. Pooled estimates were calculated from study-level resistance proportions using an inverse-variance random-effects model with the DerSimonian–Laird estimator. Total tested represents the descriptive sum of bacterial isolates tested for the corresponding antimicrobial agent and does not represent the number of tracheal aspirate specimens or patients. These totals were not used as single aggregated denominators in the random-effects models. τ^2^ is reported on the Freeman–Tukey transformed scale. *Enterobacter* spp. data were excluded from the quantitative synthesis because species-level identification was inconsistently reported.

**Table 3:** Meta-regression analyses exploring sources of heterogeneity in pooled meropenem resistance estimates.

Pathogen	Moderator	k	β	SE	95% CI	*p*	Residual I^2^
*A. baumannii*	Study mid-year	13	0.0120	0.0067	−0.0011 to 0.0251	0.072	68.5%
	Guideline	11	0.1341	0.0424	0.0511 to 0.2171	0.002 *	45.7%
*P. aeruginosa*	Study mid-year	10	−0.0184	0.0186	−0.0549 to 0.0180	0.322	91.2%
	Guideline	9	−0.1413	0.1386	−0.4130 to 0.1305	0.308	85.4%
*K. pneumoniae*	Study mid-year	9	0.0445	0.0197	0.0060 to 0.0831	0.024 *	90.3%
	Guideline	8	0.0841	0.1796	−0.2679 to 0.4361	0.640	88.9%

* *p* < 0.05. SE: standard error; CI: confidence interval.

## Data Availability

The study-level data and complete R code used to reproduce the meta-analysis are available in Supplementary Files S1 and S2.

## Supplementary Materials

The following supporting information can be downloaded at: https://www.mdpi.com/article/10.3390/biomedicines14102196/s1, Table S1: PRISMA Checklist. Table S2: Study-level characteristics, microbiological methods, and analytical units of the included studies. File S1: Complete R code used for the meta-analysis. File S2: Study-level analytical dataset used to reproduce the pooled estimates and forest plots.

## Appendix A

**Table A1:** Search terms.

Database	Search Terms
PubMed/MEDLINE	(“Tracheal Aspirate” OR “Endotracheal Aspirate” OR tracheal aspirate* OR endotracheal aspirate* OR ETA OR deep tracheal aspirate*) AND (“Drug Resistance, Bacterial” OR antimicrobial resistance OR antibiotic resistance OR multidrug resistance OR MDR OR XDR OR susceptibility) AND (“Gram-negative bacteria” OR *Acinetobacter baumannii* OR *Pseudomonas aeruginosa* OR *Klebsiella pneumoniae* OR *Escherichia coli* OR *Enterobacter* spp.) AND (ICU OR intensive care unit* OR ventilator-associated pneumonia OR VAP) AND (Turkey OR Türkiye)
Scopus	TITLE-ABS-KEY (“tracheal aspirate*” OR “endotracheal aspirate*” OR ETA OR “deep tracheal aspirate*”) AND TITLE-ABS-KEY (“antibiotic resistance” OR “antimicrobial resistance” OR MDR OR XDR OR susceptibility) AND TITLE-ABS-KEY (“*Acinetobacter baumannii*” OR “*Pseudomonas aeruginosa*” OR “*Klebsiella pneumoniae*” OR “*Escherichia coli*” OR *Enterobacter*) AND TITLE-ABS-KEY (ICU OR “intensive care unit” OR VAP OR “ventilator-associated pneumonia”) AND TITLE-ABS-KEY (Turkey OR Türkiye)
Web of Science	TS = (“tracheal aspirate*” OR “endotracheal aspirate*” OR ETA OR “deep tracheal aspirate*”) AND TS = (“antibiotic resistance” OR “antimicrobial resistance” OR MDR OR XDR OR susceptibility) AND TS = (“*Acinetobacter baumannii*” OR “*Pseudomonas aeruginosa*” OR “*Klebsiella pneumoniae*” OR “*Escherichia coli*” OR *Enterobacter*) AND TS = (ICU OR “intensive care unit” OR VAP OR “ventilator-associated pneumonia”) AND TS = (Turkey OR Türkiye)
Embase	(‘tracheal aspirate’/exp OR ‘endotracheal aspirate’/exp OR tracheal aspirate*:ti,ab OR endotracheal aspirate*:ti,ab OR ETA:ti,ab) AND (‘drug resistance, bacterial’/exp OR ‘antimicrobial resistance’/exp OR resistance:ti,ab OR MDR:ti,ab OR XDR:ti,ab OR susceptibility:ti,ab) AND (‘*Acinetobacter baumannii*’/exp OR ‘*Pseudomonas aeruginosa*’/exp OR ‘*Klebsiella pneumoniae*’/exp OR ‘*Escherichia coli*’/exp OR enterobacter*:ti,ab) AND (‘intensive care unit’/exp OR ICU:ti,ab OR ‘ventilator associated pneumonia’/exp OR VAP:ti,ab) AND (Turkey:ti,ab OR Türkiye:ti,ab)
Turk Medline	(“trakeal aspirat” OR “endotrakeal aspirat” OR “derin trakeal aspirat” OR “tracheal aspirate” OR “endotracheal aspirate”) AND (“antibiyotik direnci” OR “antimikrobiyal direnç” OR “antibiyotik duyarlılığı” OR “antimicrobial resistance”)
Google Scholar	“trakeal aspirat” “antibiyotik direnci” Türkiye; “endotrakeal aspirat” “antibiyotik duyarlılığı” Türkiye; “tracheal aspirate” “antimicrobial resistance” Turkey; “endotracheal aspirate” “antibiotic resistance” Turkey

* Denotes truncation in database searches, allowing retrieval of words with different endings (e.g., aspirate* retrieves aspirate and aspirates).
