# Supplementary material for: Antimicrobial Resistance Patterns Among Gram-Negative Bacterial Isolates Recovered from Tracheal Aspirate Cultures in Türkiye: A Systematic Review and Meta-Analysis
Source: Biomedicines. 2026 Sep 28;14(10):2196. doi: 10.3390/biomedicines14102196 (PMC13701897; doi:10.3390/biomedicines14102196)
Supplement: Supplementary file 1 [file biomedicines-14-02196-s001.zip › File S1.pdf]

```
# Complete reproducibility code for Reviewer Comment 3
# Requires: metafor (tested with current CRAN releases)
# Input: study_level_data.csv
# Required columns:
#   study, pathogen, antibiotic, events, total, year, guideline
# `events` = resistant isolates; `total` = isolates tested.
# No aggregated isolate-level row should be supplied.

rm(list = ls())
options(stringsAsFactors = FALSE)

if (!requireNamespace("metafor", quietly = TRUE)) {
  install.packages("metafor", repos = "https://cloud.r-project.org")
}
library(metafor)

# ---------- 1. Read and validate study-level data ----------
# Replace with the final submitted supplementary-data filename.
data_file <- "study_level_data.csv"
dat <- read.csv(data_file, check.names = FALSE)

required <- c("study", "pathogen", "antibiotic", "events", "total",
              "year", "guideline")
missing_cols <- setdiff(required, names(dat))
if (length(missing_cols) > 0) {
  stop("Missing required columns: ", paste(missing_cols, collapse = ", "))
}

if (anyNA(dat[, c("study", "pathogen", "antibiotic", "events", "total")])) {
  stop("Missing values are present in required analysis columns.")
```

```r
}
if (any(dat$events < 0 | dat$events > dat$total)) {
  stop("Each row must satisfy 0 <= events <= total.")
}
if (any(dat$total <= 0)) {
  stop("All denominators must be positive.")
}
if (any(dat$events != round(dat$events) | dat$total != round(dat$total))) {
  stop("events and total must be integer counts.")
}

# This check prevents accidental inclusion of an already aggregated row.
# Delete only if a genuine study is named 'Aggregated'.
if (any(grepl("aggregat|pooled|total", dat$study, ignore.case = TRUE))) {
  warning("Check study labels for accidental aggregated rows.")
}

# ---------- 2. One pathogen-antibiotic meta-analysis ----------
# Freeman-Tukey double-arcsine transformation:
#   metafor::escalc(measure = 'PFT')
# Random-effects weighting:
#   1 / (vi + tau^2)
# Between-study variance:
#   DerSimonian-Laird, method = 'DL'
# Back-transformation:
#   Miller inverse PFT using harmonic mean of study denominators.

fit_one <- function(d, make_plot = FALSE, plot_file = NULL) {
  if (nrow(d) < 2) {
    stop("At least two studies are required for a pooled analysis.")
  }
```

```
# add = 0 is explicit; PFT handles 0 and 1 proportions without a
# continuity correction in metafor.
es <- metafor::escalc(
  measure = "PFT",
  xi = events,
  ni = total,
  data = d,
  add = 0,
  to = "none"
)

# Random-effects model on the transformed scale.
fit <- metafor::rma(
  yi = yi,
  vi = vi,
  data = es,
  method = "DL"
)

# Back-transform each study using its own denominator.
study_bt <- data.frame(
  study = es$study,
  events = es$events,
  total = es$total,
  proportion = es$events / es$total,
  yi = es$yi,
  vi = es$vi,
  ci.lb = metafor::transf.ipft(
    es$yi - qnorm(0.975) * sqrt(es$vi),
    ni = es$total
```

```
  ),
  ci.ub = metafor::transf.ipft(
    es$yi + qnorm(0.975) * sqrt(es$vi),
    ni = es$total
  )
)
study_bt$estimate <- metafor::transf.ipft(es$yi, ni = es$total)

# Back-transform pooled estimate and CI with harmonic mean of ni.
pooled <- predict(
  fit,
  transf = metafor::transf.ipft.hm,
  targs = list(ni = es$total)
)

out <- data.frame(
  pathogen = d$pathogen[1],
  antibiotic = d$antibiotic[1],
  k = fit$k,
  total_isolates_descriptive = sum(d$total),
  resistant_isolates_descriptive = sum(d$events),
  pooled_resistance = as.numeric(pooled$pred),
  ci_lb = as.numeric(pooled$ci.lb),
  ci_ub = as.numeric(pooled$ci.ub),
  tau2 = as.numeric(fit$tau2),
  I2_percent = as.numeric(fit$I2),
  Q = as.numeric(fit$QE),
  Q_df = as.numeric(fit$k - fit$p),
  Q_p = as.numeric(fit$QEp),
  stringsAsFactors = FALSE
)
```

```
if (make_plot) {
  if (!is.null(plot_file)) {
    grDevices::pdf(plot_file, width = 8.5, height = 7)
    on.exit(grDevices::dev.off(), add = TRUE)
  }

  # Plot directly on the original proportion scale. The study-specific
  # point estimates and CIs have already been inverse transformed with
  # study-specific denominators, so no common n is incorrectly used.
  safe_slab <- iconv(as.character(study_bt$study), from = "UTF-8",
                     to = "ASCII//TRANSLIT", sub = "")
  metafor::forest(
    study_bt$estimate,
    ci.lb = study_bt$ci.lb,
    ci.ub = study_bt$ci.ub,
    slab = safe_slab,
    alim = c(0, 1),
    at = seq(0, 1, by = 0.2),
    xlab = "Resistance proportion",
    header = c("Study", "Proportion [95% CI]"),
    refline = NA,
    psize = 1,
    digits = 2,
    ylim = c(-2, fit$k + 4)
  )
  metafor::addpoly(
    x = pooled$pred,
    ci.lb = pooled$ci.lb,
    ci.ub = pooled$ci.ub,
    row = -1,
```

```
      mlab = "Random-effects model (DerSimonian-Laird)",
      annotate = TRUE,
      digits = 2
    )
    graphics::title(
      main = paste(d$pathogen[1], "-", d$antibiotic[1]),
      cex.main = 0.95
    )
  }

  list(model = fit, effect_sizes = es, study_original_scale = study_bt,
       pooled_original_scale = pooled, summary = out)
}

# ---------- 3. Run every pathogen-antibiotic combination ----------
groups <- split(dat, interaction(dat$pathogen, dat$antibiotic,
                                 drop = TRUE, sep = "|||"))
groups <- groups[vapply(groups, nrow, integer(1)) >= 2]

results <- vector("list", length(groups))
plots_dir <- "revised_forest_plots"
if (!dir.exists(plots_dir)) dir.create(plots_dir)

for (i in seq_along(groups)) {
  g <- groups[[i]]
  safe_name <- gsub("[^A-Za-z0-9]+", "_",
                    paste(g$pathogen[1], g$antibiotic[1], sep = "_"))
  results[[i]] <- fit_one(
    g,
    make_plot = TRUE,
    plot_file = file.path(plots_dir, paste0(safe_name, ".pdf"))
```

```
  )
}

reconciled_table <- do.call(rbind, lapply(results, `[[`, "summary"))
rownames(reconciled_table) <- NULL

# Percentage format for direct comparison with manuscript Table 2.
reconciled_table$pooled_percent <- 100 * reconciled_table$pooled_resistance
reconciled_table$ci_lb_percent <- 100 * reconciled_table$ci_lb
reconciled_table$ci_ub_percent <- 100 * reconciled_table$ci_ub

write.csv(reconciled_table, "reconciled_meta_analysis_results.csv",
          row.names = FALSE, na = "")

# ---------- 4. Optional explicit checks for the five analyses in Figure 2 ----------
check_fig2 <- subset(
  reconciled_table,
  (pathogen == "A. baumannii" & antibiotic == "MEM") |
  (pathogen == "P. aeruginosa" & antibiotic == "MEM") |
  (pathogen == "K. pneumoniae" & antibiotic == "MEM") |
  (pathogen == "Enterobacter spp." & antibiotic == "MEM") |
  (pathogen == "E. coli" & antibiotic == "MEM")
)
print(check_fig2)

# ---------- 5. Optional meta-regression, using the same fitted models ----------
# This reproduces the manuscript's moderators on the transformed scale.
meta_regression_one <- function(d, moderator) {
  es <- metafor::escalc(measure = "PFT", xi = events, ni = total,
                        data = d, add = 0, to = "none")
  fit <- metafor::rma(yi, vi, mods = as.formula(paste("~", moderator)),
```

```
                    data = es, method = "DL")
  fit
}

# Example:
# d <- subset(dat, pathogen == "K. pneumoniae" & antibiotic == "MEM")
# print(meta_regression_one(d, "year"))
# print(meta_regression_one(d, "guideline"))

# Session information for reproducibility.
writeLines(capture.output(sessionInfo()), "sessionInfo_comment3.txt")

message("Completed. Review reconciled_meta_analysis_results.csv, revised_forest_plots/, and
sessionInfo_comment3.txt.")
```
