# Supplementary material for: Antimicrobial Resistance Patterns Among Gram-Negative Bacterial Isolates Recovered from Tracheal Aspirate Cultures in Türkiye: A Systematic Review and Meta-Analysis
Source: Biomedicines. 2026 Sep 28;14(10):2196. doi: 10.3390/biomedicines14102196 (PMC13701897; doi:10.3390/biomedicines14102196)
Supplement: Supplementary file 1 [file biomedicines-14-02196-s001.zip › Table S2.pdf]

Table S2. Study-level characteristics, microbiological methods, and analytical units of the included studies.

Study	Study period	Patients, n	Tracheal aspirate specimens, n	Culture-positive specimens, n	Total isolates/ microorganisms, n	Gram-negative isolates included, n	Patient population	Sampling indication	VAP/HAP criteria	Specimen collection	Handling of repeated isolates	Culture threshold	Identification method	AST method	Notes
Safak et al., 2007	2004–2006	455	455	NR	216	128	Mechanically ventilated ICU patients	Suspected pneumonia	VAP onset 48–72 h after intubation	ETA; Lukens container; qualitative/quantitative culture	NR	2004 qualitative; 2005–2006 quantitative >10^5 CFU/mL	CM	mini-API and Phoenix	Target total is the five prespecified Gram-negative groups.
Ulug et al., 2011	2003–2005	72	NR	NR	84	54	ICU patients ventilated >48 h with VAP	Suspected VAP	Clinical–radiological–microbiological criteria	ETA; Lukens tube; quantitative culture	NR	≥10^5 CFU/mL	CM	KB/DD	Twelve patients had polymicrobial growth; 63 Gram-negative isolates overall.
Kume and Demirci, 2012	2008–2010	125	NR	NR	177	175	Intubated or tracheostomized ICU patients	Routine lower respiratory tract sampling	ND	Tracheal aspirate; sterile catheter; qualitative culture	Repeated isolates with the same organism and susceptibility pattern were excluded (n=16).	NR	CM	KB/DD	Initial 193 non-fermenters; 177 analyzed.
Atasoy et al., 2014	2012–2013	NR	NR	NR	212	102	Hospital patients with A. baumannii; TA	Routine clinical sampling	ND	Collection method NR; routine culture	NR	NR	VITEK 2	VITEK 2	212 *A. baumannii* isolates from all specimen

							subgroup analyzed								types; 102 were tracheal aspirate isolates.
Dede et al., 2014	2010–2011	165	192	NR	280	220	ICU patients ventilated >48 h with suspected VAP	Suspected VAP	VAP onset ≥48 h after mechanical ventilation	DTA; closed-system aspiration; culture	NR	NR	VITEK 2	VITEK 2	
Karakece et al., 2014	2011–2012	NR	856	304	304	257	ICU patients with submitted ETA specimens	Routine ICU sampling	VAP onset ≥48 h after mechanical ventilation; inclusion criteria NR	ETA; qualitative culture	Repeated tracheal aspirate growth was excluded.	NR	VITEK 2	VITEK 2	The study reported 17 *E. coli* isolates; however, these isolates were not included in the meta-analysis dataset because antimicrobial susceptibility results were not reported.
Ozunel et al., 2014	2012–2013	217	238	77	81	81	Mechanically ventilated anesthesia ICU	VAP evaluation	VAP onset ≥48 h after intubation	DTA; collection method NR; culture	NR	NR	VITEK 2	VITEK 2	Four specimens yielded both A. baumannii

							patients with VAP								and *P. aeruginosa*.
Aydemir et al., 2016	2015 – 2016	350	350	130	141	110	Mechanically ventilated ICU patients	Suspected LRTI	ND	ETA; sterile catheter; culture	NR	NR	VITEK 2	VITEK 2	
Sagmak-Tartar et al., 2018	2016 – 2017	NR	1470	620	626	553	ICU patients with submitted ETA cultures	Suspected VAP	ND	ETA; sterile catheter; quantitative culture	Repeat specimens yielding the same organism were excluded.	≥100,000 CFU/mL	VITEK 2	KB/DD	Total included 584 Gram-negative, 36 Gram-positive and 6 Candida isolates.
Koca, 2019	2017 – 2018	103	205	113	113	88	Adult ICU patients with submitted ETA cultures	Routine ICU sampling	ND	ETA; sterile catheter; quantitative culture	Same organism from different tracheal aspirates of one patient was excluded.	≥100,000 CFU/mL	VITEK	VITEK	Ten samples had double growth.
Arici et al., 2020	2016 – 2018	NR	NR	NR	385	337	ICU patients with clinically significant Gram-negative ETA isolates	Routine ICU sampling	ND	ETA; collection method NR; quantitative culture	Repeated isolates were excluded.	≥10^5 CFU/mL	MALDI-TOF/VITEK 2	VITEK 2	

Caskurlu et al., 2020	2014 – 2018	NR	5201	2725	2725	1907	Patients with pure, clinically significant TA growth	Suspected pneumonia/LRTI	ND; nosocomial infection onset >48 h	TA; sterile catheter; quantitative culture	Only the first tracheal aspirate isolate per patient was included.	≥100,000 CFU/mL	VITEK 2	VITEK 2	
Duran et al., 2021	2015 – 2019	NR	NR	NR	773	505	ICU patients with A. baumannii; ETA subgroup analyzed	Routine ICU sampling	ND	ETA; collection method NR; routine culture	First isolate from the same patient and same clinical sample was included.	NR	BD Phoenix	BD Phoenix	773 *A. baumannii* across all specimen types; 505 were tracheal aspirate isolates.
Ayvalik et al., 2022	2018 – 2021	NR	1031	458	458	356	ICU patients with significant ETA growth	Routine ICU sampling	ND	ETA; collection method NR; quantitative culture	Repeated growth from the same patient was excluded.	NR	BD Phoenix	BD Phoenix	458 isolates: 413 Gram-negative and 45 Gram-positive.
Genc Bahce et al., 2022	2019-2020	1573	NR	140	206	142	General and COVID-19 ICU patients with LRTI	Suspected LRTI	ND	ETA; Lukens tube; quantitative culture	For repeated recovery of the same organism from one patient, only the first isolate was used.	≥10^5 CFU/mL	VITEK 2	VITEK 2	

Gudul Havuz et al., 2022	2019–2020	74	NR	NR	74	74	ICU patients with A. baumannii from ETA; stay ≥48 h	Healthcare-associated infection evaluation	Infection onset ≥48 h; no specific VAP/HAP definition	ETA; sterile catheter; culture	First growth per patient was included.	NR	VITEK 2	VITEK 2	
Oguz Mizrakci, 2022	2019–2020	NR	288	140	140	116	ICU patients with submitted DTA cultures	Routine ICU sampling	ND	DTA; sterile catheter; quantitative culture	Repeated isolates were excluded.	NR	CM	KB/DD	Source Table 1 and Table 2 disagree on *P. aeruginosa* and *K. pneumoniae* denominators; several percentages are incompatible with stated n.
Semet, 2025	2022	215	NR	NR	263	231	Adult medical/surgical ICU patients; stay ≥48 h and significant ETA growth	Routine ICU sampling	ND; ICU stay ≥48 h required	ETA; collection method NR; quantitative culture	NR	≥100,000 CFU/mL	Conventional	KB/DD and E-test	

AST, antimicrobial susceptibility testing; BD, Becton Dickinson; CFU/mL, colony-forming units per milliliter; CLSI, Clinical and Laboratory Standards Institute; CM, conventional methods; DTA, deep tracheal aspirate; ETA, endotracheal aspirate; EUCAST, European Committee on Antimicrobial Susceptibility Testing; HAP, hospital-

acquired pneumonia; ICU, intensive care unit; KB/DD, Kirby–Bauer disk diffusion; LRTI, lower respiratory tract infection; MALDI-TOF MS, matrix-assisted laser desorption/ionization time-of-flight mass spectrometry; NCCLS, National Committee for Clinical Laboratory Standards; ND, not defined; NR, not reported; R, resistant; S, susceptible; TA, tracheal aspirate; VAP, ventilator-associated pneumonia.
